# Comparative Analysis of Porcine Follicular Fluid Proteomes of Small and Large Ovarian Follicles

**DOI:** 10.3390/biology9050101

**Published:** 2020-05-17

**Authors:** Victor M. Paes, José R. de Figueiredo, Peter L. Ryan, Scott T. Willard, Jean M. Feugang

**Affiliations:** 1 Department of Animal and Dairy Sciences, Mississippi State University, Starkville, MS 39759, USA; macedovictor_ef@yahoo.com.br (V.M.P.); Ryan@provost.msstate.edu (P.L.R.); swillard@cals.msstate.edu (S.T.W.); 2 Laboratory of Manipulation of Oocyte and Preantral follicles, State University of Ceará, CEP, 60740 903 Fortaleza, Brazil; figueiredo.lamofopa@gmail.com

**Keywords:** oocyte maturation, follicle growth, pig fertilization

## Abstract

Ovarian follicular fluid is widely used for in vitro oocyte maturation, but its in-depth characterization to extract full beneficial effects remains unclear. Here, we performed both shotgun (nanoscale liquid chromatography coupled to tandem mass spectrometry or nanoLC-MS/MS) and gel-based (two dimension-differential in-gel electrophoresis or 2D-DIGE) proteomics, followed by functional bioinformatics to compare the proteomes of follicular fluids collected from small (<4 mm) and large (>6–12 mm) follicles of pig ovaries. A total of 2321 unique spots were detected with the 2D-DIGE across small and large follicles, while 2876 proteins with 88% successful annotations were detected with the shotgun approach. The shotgun and 2D-DIGE approaches revealed about 426 and 300 proteins that were respectively common across samples. Six proteins detected with both technical approaches were significantly differently expressed between small and large follicles. Pathways such as estrogen and PI3K-Akt signaling were significantly enriched in small follicles while the complement and coagulation cascades pathways were significantly represented in large follicles. Up-regulated proteins in small follicles were in favor of oocyte maturation, while those in large follicles were involved in the ovulatory process preparation. Few proteins with potential roles during sperm–oocyte interactions were especially detected in FF of large follicles and supporting the potential role of the ovarian FF on the intrafallopian sperm migration and interaction with the oocyte.

## 1. Introduction

During follicle development, in addition to somatic cells proliferation and oocyte growth, there is the appearance of an antrum cavity. This essential cavity is filled with a follicular fluid (FF) rich in blood plasma molecules and secretions of follicle cells [1], constituting an important microenvironment for normal folliculogenesis and oocyte development [2].

Numerous studies have investigated the FF composition to find key molecules that could support oocyte maturation [3,4,5,6,7,8]. Considering the progress achieved thus far, various but still unknown, molecules of FF that contribute to normal folliculogenesis have shown be follicle size-dependent [9,10,11]. Regarding in vitro maturation of oocytes, it is clearly established that follicles with small diameters mainly contain oocytes achieving highest nuclear maturation with first polar body exclusion but limited cytoplasmic maturation (cumulus cell-expansion and embryo production) [12,13,14,15,16,17]. In contrast, growing follicles contain oocytes with greater capabilities for both nuclear and cytoplasmic maturation [9,10,18,19,20]. These evolving beneficial effects in growing follicles may be reflected on the changing composition of the FF throughout folliculogenesis, reaching optimum developmental biomolecule interactions to enhance oocyte quality in larger follicles [14,21,22]. There is a widely use of FF to mature pig oocytes in vitro [20,21,23], with FF derived from large antral follicles enhancing maturation of oocytes from small follicles [24] and subsequent embryo development [2]. FF is a complex biofluid which critical components and molecular events accounting for developmental potential acquisition are still unclear.

High-throughput proteomics are sophisticated tools used to investigate the protein profile of biological material and complex biofluids, such as FF [25]. Both the classic gel-based proteomic and the bottom-up gel-free methods can be used to investigate the protein content of FF samples. While the gel-free known as shotgun approach allows for the detection of many proteins, its combination with the gel-based approach could afford the discovery of greater number of proteins in comparative studies [26].

Proteomics approaches have been used in comparative or profiling studies to investigate the FF of women [27], sows [28,29], mares [30,31], cows [3], and goats [7]. These studies revealed a higher number of proteins in the FF, but only few (e.g., anti-oxidative proteins, growth factors, and hormones) are routinely supplemented to culture media to enhance developmental competence of in vitro matured oocytes [21,32,33]. In comparison to individually added components to culture media, the FF generally offers better developmental effects suggesting possible concerted actions to specific biological processes. At present, little information is available on the molecular control of follicle function throughout folliculogenesis.

The identification of involved proteins and their biological significance may be essential to create a well-appropriated culture medium to improve assisted reproduction outcomes. Therefore, we conducted both gel-free (nanoLC-MS/MS) and gel-based (2D-DIGE) proteomics to compare the proteomes of FF derived from small (<4 mm or SFF) and large (>6–12 mm; LFF) porcine follicles to identify key proteins involved in early and late follicle growth.

## 2. Materials and Methods

### 2.1. Follicle Categorization, Follicular Fluids Aspirations, Estradiol (E2), and Protein Assays

A total of 120 sow ovaries were harvested post-mortem at a local abattoir, transported to the laboratory on ice within 2 h, and washed with a 0.9% (v/v) NaCl solution containing 1 µg/mL antibiotics (Penicillin/Streptomycin). Ovaries devoid of any active corpus luteum were selected for follicles dissection, followed by their grouping as small (<4 mm) or large (>6–12 mm) diameters. Follicles with colorless and homogenous texture were selected for follicular fluid (FF) aspiration using appropriated needles fixed to syringes. In large follicles, FF were aspirated from individual follicles (~15 ovaries per harvest) while in small follicles, FF samples were pools of several follicles of individual ovaries (~15 per harvest). All FF samples were centrifuged (1600× *g*; 5 min) at 4 °C to eliminate cells and other debris. Derived supernatants were collected and kept on ice. Aliquots (100 µL) were taken for protein and estradiol (E2) assays. Follicular fluids with highest E2 concentrations in each small (SFF) and large (LFF) follicles were selected and referred to as small non-atretic (SNA) and large non-atretic (LNA) samples, respectively. In each category, samples with comparable E2 values were mixed to constitute three pools per ovary harvest. All procedures were performed as previously reported [34], with a total of four independent harvests performed for the study. Twelve pools of each SNA and LNA group were constituted and all pools were subsequently stored at −80 °C until proteomic analyses.

### 2.2. Proteomic Analyses

Both gel-free and gel-based methods were used to run three pools of SNA and LNA samples. These proteomic samples corresponded to mixtures of frozen-thawed pools of comparable E2 concentrations of each abovementioned harvest (*n* = 4). All six constituted pools were subjected to individual proteomic runs/analyses.

The gel-free or shotgun (NanoSpray LC/MS, Thermo Fisher Scientific, Waltham, MA, USA) method was used as previously described [35]. Briefly, FF protein samples (50 µg) were precipitated (50% acetone-trichloroacetic acid), digested (trypsin), desalted (Strong Cation Exchange (SCE) Microtrap wash: 2% acetonitrile or ACN-Elution: 90% ACN), dried (vacuum centrifugation), cleaned (2 X washes through SCE: 5 mM sodium phosphate, 25% ACN, pH3-Elution: 5 mM sodium phosphate, 25% ACN, 0.25 M potassium chloride, pH3), dried, and resulting salt crystals and peptides were resuspended in 5% ACN (20 µL) and transferred to a low retention autosampler vial for deconvolution via reverse phase, high-pressure liquid chromatography (BioBasic C18 reversed phase column). Samples were flushed for 20 min with 5% ACN to remove salts. Peptides were separated with 655 min nano-HPLC method, consisting of 620 min (gradient from 5% to 50% ACN), followed by a 20 min (wash with 95% ACN), and 15 min (equilibration with 5% ACN). All solvents contained 0.1% formic acid as a proton source for pH adjustment. Peptides were ionized (Thermo Finnigan Nanospray ionization, type I source) at a high-voltage (1.85 kV) using a t-connector with a gold electrode in contact with eh HPLC solvent and 8 µm interval diameter silica tips (New Objective FS360-75-8-N-20-C12). A Thermo LCQ DECA XP Plus ion trap mass spectrometer was used to collect data over the 655 min duration of each HPLC run, and precursor mass scans were used as previously described [35]. All materials and equipment were obtained from Thermo Fisher Scientific (Waltham, MA, USA).

The gel-based method was performed through a two dimension-differential in-gel electrophoresis (2D-DIGE), followed by a mass spectroscopy protein identification. Frozen-thawed FF samples were subjected to analyses (Applied Biomics, Inc, Hayward, CA, USA). Briefly, sample proteins were extracted with 200 µL 2D lysis buffer (2 M thiourea, 7 M urea, 4% CHAPS, 30 mM Tris-HCl, pH 8.8). Mixtures were sonicated, agitated (30 min), centrifuged (16,000 rpm; 30 min at 4 °C), and supernatants were collected for protein assay (Bio-Rad protein assay) followed by a dilution (2-D cell lysis buffer) to 5 µg protein /µL. Aliquots of 30 µg were subjected to minimal CyDye labeling with 1.0 µL of 0.2 nmol/µL CyDye (Cy2, Cy3, and Cy5) of 30 min on ice, under dark. Lysine (1.0 µl of 10 mM) was added to each sample and incubated (on ice, for 15 min under dark). Thereafter, equal amounts of samples labeled with each Cydye were mixed and 2 X 2-D sample buffer (8 M urea, 4% CHAPS, 20 mg/mL DTT, 2% pharmalytes and trace amount of bromophenol blue) was added, together with 100 µL destreak solution and approximately 236 µL Rehydration buffer (7 M urea, 2 M thiourea, 4% CHAPS, 20 mg/mL DTT, 1% pharmalytes and trace amount of bromophenol blue) to reach a volume of 350 µL. Three independent samples were prepared for each Cydye labeling and mixtures were spun before loading into the strip holder for Isoelectric focusing (IEF), using 18 cm IPG strips that were placed faced down and covered by mineral oil (1.5 mL). The IEF was run under dark at 20 °C, according to the manufacturer’s recommendation (Amersham BioSciences, Piscataway, NJ, USA). Thereafter, protein loaded-IPGs were successfully equilibrated for 15 min with buffer 1 (50 mM Tris-HCl, pH 8.8, containing 6 M urea, 30% glycerol, 2% SDS, a trace amount of bromophenol blue and 10 mg/mL DTT) and 10 min with buffer 2 (50 mM Tris-HCl, pH 8.8, containing 6 M urea, 30% glycerol, 2% SDS, a trace amount of bromophenol blue and 45 mg/mL iodacetamide) under slow shaking. Subsequently, equilibrated loaded-IPG strips were rinsed once in the running buffer and transferred into the SDS-Gel (12% SDS-gel prepared using low fluorescent glass plates). The IPGs were sealed with 0.5% (w/v) agarose solution, and gels were run at 15 °C, immediately followed by gel image scanning (Typhoon TRIO; Cytiva, Marlborough, MA, USA). Scanned images were evaluated (Image QuantTL software; Cytiva) and gels were subjected to in- and cross-gel analyses of protein differential expression expressed by ratio change (DeCyder software version 6.5; Cytiva). Protein spots of interest were picked up (Ettan Spot Picker; Cytiva) based on in-gel analyses and spot picking design by DeCyder software. After 2–3 washes and in-gel protein digestion (Trypsin Gold; Promega, Madison, WI, USA), samples were desalted (Zip-tip C18; Millipore, Billerica, MA, USA), and obtained peptides were eluted with a matrix solution (α-cyano-4-hydroxycinnamic acid, 5 mg/mL in 50% acetonitrile, 0.1% trifluoroacetic acid, 25 mM ammonium bicarbonate) and spotted on the MALDI plate. MALDI-TOF (MS) and TOF/TOF (tandem MS/MS) were performed on a TOF/TOF 5800 mass spectrometer (AB Sciex, Framingham, MA, USA), and mass spectra were acquired in reflectron positive ion mode, averaging 2000 laser shots per spectrum. TOF/TOF tandem MS fragmentation spectra were acquired for each sample, averaging 2000 laser shots per fragmentation spectrum on each of the 10 most abundant ions present in each sample (excluding trypsin autolytic peptides and other known background ions).

### 2.3. Database Search, Protein Function, and Pathway Identification

Resulting peptide mass and associated fragmentation spectra were submitted to GPS Explorer version 3.5 equipped with the MASCOT search engine (Matrix science) to search the database of National Center for Biotechnology Information non-redundant (NCBInr). Searches were performed without constraining protein molecular weight or isoelectric point, with variable carbamidomethylation of cysteine and oxidation of methionine residues, and with one missed cleavage allowed in the search parameters. Peptides without corresponding proteins in NCBInr was subjected to *Sus scrofa* ENSEMB for potential matches. Proteins that scored greater than 95% on either protein (C.I. %) or Ion (C.I. %) were considered significant and retained for further analyses.

Venn diagrams were generated for proteins distribution across follicle sizes (http://bioinformatics.psb.ugent.be/cgi-bin/liste/Venn/calculate_venn.htpl). Functional annotations (biological process, cellular localization, and molecular function) were performed using the Agbase platform (https://agbse.arizna.edu/) and Single Enrichment Analysis (SEA) of gene ontologies (GO), pathways, and protein-to-protein interactions (PPI) were assessed with STRING (www.string-db.org).

### 2.4. Statistical Analyses

Estradiol and protein concentrations were statistically analyzed with One-way ANOVA, followed by the Fishers’ LSD test. Search results for peptide marches were filtered with decoy based, and proteins corresponding to at least three peptides with (Benjamini–Hochberg correction) probability of 0.05 or less were retained as indicative of the confidence in protein identification and relative expression [36]. Bioinformatics analyses were performed using the default setting of each online software and protein association networks were obtained with the highest confidence interaction score (>0.9). The Fischer’s Exact Test with Multiple Testing Correction of Benjamini–Hochberg false discovery rates (FDR) was set at 5% threshold, indicative of probability that the association of proteins with given biological function or pathway was not due to random chance.

## 3. Results

### 3.1. Estradiol (E2) and Protein Concentration

Regardless of follicle size, the E2 levels significantly varied in FF of both small and large follicles. The FF samples containing highest E2 levels in each follicle category size were considered non-atretic (NA) and were used for further analyses. The average E2 levels in selected large non-atretic (LNA) samples was significantly higher than that of small non-atretic (SNA) (289.9 ± 37 ng/mL vs. 26.11 ± 15 ng/mL; *p* < 0.05). However, the total protein concentrations in both samples remained comparable (2.83 ± 0.6 µg/mL vs. 2.85 ± 0.6 µg/mL, for SNA vs. LNA, respectively; *p* > 0.05).

### 3.2. Gel-Free (Shotgun) Proteome Identification and Functional Analyses

**Figure 1:**
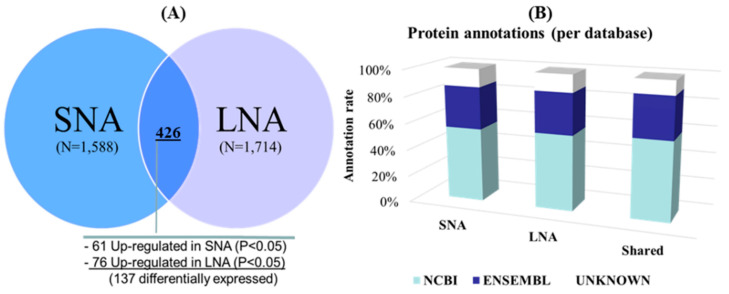
Total protein detection (**A**) and annotations (**B**). Venn diagram of porcine follicular fluid was constructed with tools available at the Bioinformatics and Evolutionary Genomics, Ghent, Belgium. SNA: small non-atretic; LNA: large non-atretic; NCBI: National Center for Biotechnology Information. P < 0.05 indicates significant difference.

Protein identification. Post-shotgun analyses revealed a total of 2876 unique proteins across samples, with 1588 (55%) in SNA and 1714 (59%) in LNA (Table S1). The Venn diagram in Figure 1A shows 426 proteins, representing 15% of unique proteins that were commonly shared between LNA and SNA samples, with 137 differentially detected (61 up-regulated in SNA and 76 in LNA; *p* < 0.05). As to both sample types, 73% (1162) and 75% (1288) of their global proteomes were specifics to SNA and LNA, respectively. Figure 1B shows the successful annotation of 88% of total detected proteins with both NCBI and ENSEMBL database repositories.

Gene ontology (GO) enrichment and reactome pathways of shared proteins. Focusing on up-regulated proteins in SNA, the functional analyses revealed no significant (FDR > 0.05) GO terms enrichment of biological functions (cellular component, CC; biological process, BP; and molecular function, MF). Contrarily with up-regulated proteins in LNA, a variety of GO terms were significantly enriched and the top 10 significant (FDR < 0.05) GO terms are summarized in Table 1.

Proteins associated with extracellular and blood microparticles were highly represented in CC category (FDR < 8 × 10^−4^). GO terms related to regulation of catalytic, endopeptidase, or hydrolase activities in the BP category were highly significantly enriched (FDR = 6.01 × 10^−6^). The activities of enzyme regulators and inhibitors, as well as the endopeptidase inhibitor were predominantly significant in MF (FDR < 3.9 × 10^−7^), while transporter activity, serine-type endopeptidase and inhibitor were up-regulated (FDR < 0.05). Reactome analyses, representing diagrams of series of interconnected molecular events, revealed numerous significantly (FDR < 0.03) enriched pathways (Table 2). Up-regulated proteins in SNA had ten and completely different pathways than that of LNA dataset, exhibiting six pathways as “platelet degranulation”, “intrinsic pathway of fibrin clot formation”, “hemostatis”, “dissolution of fibrin clot”, “common pathway of fibrin clot formation”, and “regulation of complement cascade”.

Gene ontology (GO) enrichment and reactome of specific proteins. Only LNA specific proteins showed significant enrichments (FDR < 0.05), limited to the molecular function category. The GO terms consisted of binding (GO:0005488; FDR < 0.03), ion binding (GO:0043167; FDR < 0.03), catalytic activity (GO:0003824; FDR < 0.04), and carbohydrate derivative binding (GO:0097367; FDR < 0.04) GO terms (Table S1). Regarding the reactome analyses, only SNA dataset had significant (FDR < 0.03) pathways comprising “metabolism of proteins”, “cell cycle”, “post-translational protein modification”, and “cell cycle, mitotic” (Table 2).

**Figure 2:**
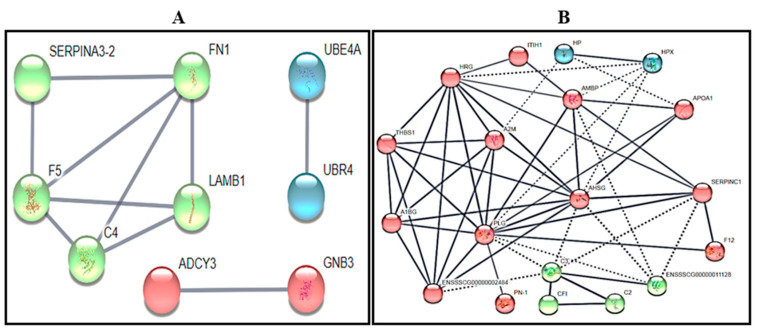
Protein-to-protein interaction networks of up-regulated proteins in small non-atretic (SNA: **A**) and large non-atretic (LNA: **B**) datasets.

Protein-to-protein interaction (PPI) networks: shared and specific proteins. Proportions of 72% (44/61) and 64% (49/76) up-regulated proteins in SNA and LNA, respectively, were successfully converted for PPI analyses and K-mean interactions (Figure 2)**,** using high confidence (0.700). Only a tendency for significant PPI enrichment was observed in the SNA dataset (Figure 2A; *p* = 0.08). However, there was a significant enrichment in LNA dataset (Figure 2B; *p* < 10^−16^) that was indicative of more interactions and biological connections (for the least) amongst proteins. Hence, the preponderant roles of alpha-2-HS-glycoprotein (AHSG), plasminogen (PLG), histidine-rich glycoprotein (HRG), AMBP, and alpha-2-macroglobulin (A2M) with at least seven connections are revealed (Figure 2B).

**Figure 3:**
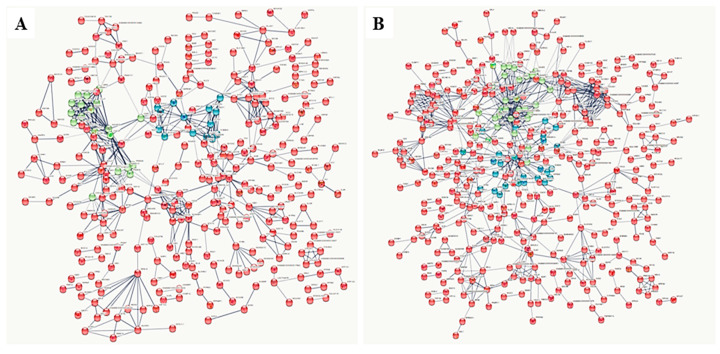
Protein–protein interaction (PPI) networks in dataset specifics to small non-atretic (SNA: **A**) and large non-atretic (LNA: **B**). The figure shows the overview dynamic pattens of PPI networks in the porcine FF during follicle growth. The LNA dataset has more interaction networks than the SNA.

Regarding specific proteins, we were able to convert 66% (780/1,182) and 67% (869/1307) for analyses in SNA and LNA, respectively. The PPI analyses revealed a tendency to a significant difference with SNA (FDR = 0.08), while a significant difference was found with LNA (FDR < 10^−16^). Changes of the global PPI patterns of both SNA and LNA datasets are shown in Figure 3A,B, respectively.

Kyoto Encyclopedia of Genes and Genomes (KEGG) pathways—Shared and specific proteins. All protein datasets (up-regulated SNA, up-regulated LNA, specific SNA, and specific LNA) revealed significantly enriched KEGG pathways (Table 3). Up-regulated proteins in SNA were characterized by significant (FDR < 0.05) enrichments of “ECM-receptor interaction”, “circadian entrainment”, and “PI3K-Akt signaling pathway” pathways. Meanwhile, “complement and coagulation cascades” and “staphylococcus aureus infection” were the only significantly (FDR = 8.17 × 10^−5^) enriched pathways in the LNA dataset. Proteins specific to SNA were enriched for “gap junction”, “estrogen signaling pathway”, and “gastric acid secretion” (*p* < 0.05), while those in LNA were associated with “protein digestion and absorption”, “focal adhesion”, and “human papillomavirus infection” (*p* < 0.05).

### 3.3. Detection of Male-Known Proteins

The shotgun approach also revealed the presence of several male-known proteins or proteins associated with fertilization. Both SNA and LNA datasets showed these proteins such as (e.g., spermatogenesis-associated protein 22, sperm specific antigen 2, and testis-specific gene 10 protein isoform X5), or were specifics to SNA (e.g., testis-expressed sequence 9 protein, testis expressed 15, sperm flagellar protein 2, spermatogenesis-associated protein5-like protein 1, and spermatid perinuclear RNA binding protein) or LNA (izumo sperm–egg fusion protein isoform X3, motile sperm domain-containing protein 2 isoform X2, sperm-associated antigen 5, and outer dense fiber of sperm tails 2).

### 3.4. Protein Identification by Gel Based (2 Dimension-Differential in-gel Electrophoresis or 2D-DIGE)

**Figure 4:**
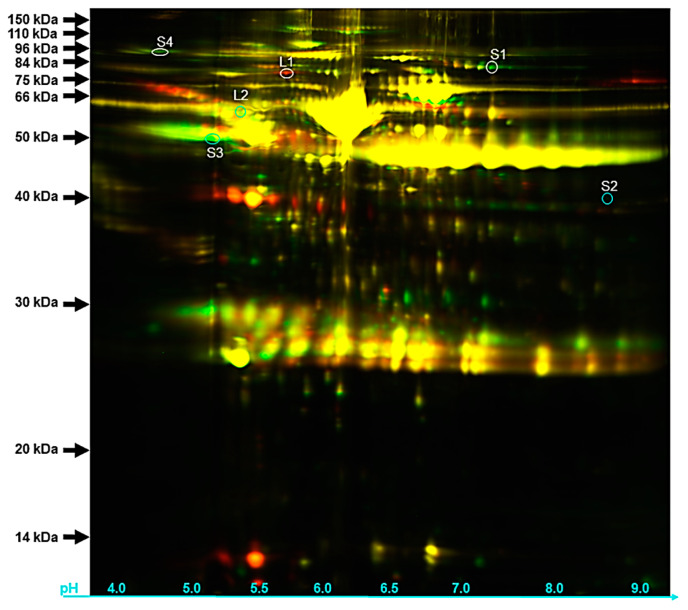
Two dimension-differential in gel electrophoresis (2D-DIGE) of protein in SNA (green spots) and LNA (red spots) samples.

The loading of 2D-DIGE gel electrophoreses with both LNA (red spots) and SNA (green spots) samples permitted the detection of about 2321 protein spots, while 300 were commonly shared and appeared as spots colored from green-to-red to yellow (Figure 4).

The selection of differentially detected spots for MS/MS analyses resulted in the identification of plasminogen precursor (spot: S1), nexin-1 precursor (spot: S2), vitronectin precursor (spot: S3), and inter-alpha-trypsin inhibitor heavy chain H2 precursor (spot: S4) that were also found in our shotgun approach (Table 4). The fluorescence signals of these protein spots were brighter than that of their SNA counterparts. On the other hand, proteins such as complement C4 precursor (spot: L1) and alpha-1 antichymotrypsin 2 precursor (spot: L2) were up-regulated in LNA shotgun dataset.

## 4. Discussion

Follicular fluid (FF) fills the antral cavity of ovarian follicles and constitutes the microenvironment of intrafollicular cells, including oocyte during follicular growth and maturation [37]. The molecular composition of the FF varies during follicle growth to likely regulate follicular cell differentiation and maturation and ovulation of the developmentally competent oocyte from the largest follicle. The present study examines the changes in protein content of FF in healthy small and large follicles of porcine ovaries.

First, we selected healthy and non-atretic FF derived from large (LNA) and small (SNA) follicle sizes containing the highest levels of estradiol, known to increase during follicle growth due to the increased activity/level of aromatase enzyme. Previous studies have reported the high intrafollicular estradiol levels as the main features of healthy follicle development in several species [35,38,39,40,41]. Furthermore, the comparable protein concentrations in selected FF samples matches with previous studies in pigs [42], cows [43], buffaloes [44], and goats [7].

Second, we profiled the protein contents of selected SNA and LNA FF using both gel free (Shotgun) and gel-based (2D-DIGE) approaches. Each technology has specific inconvenient and advantages that have been reported in previous study [27]. In the present study, we used the same batches of samples to obtain a larger pool of proteins through the shotgun method as compared to the 2D-DIGE (2876 unique proteins versus 2321 protein spots, respectively). This finding clearly demonstrated a limitation of the gel-based system that agrees with previous studies comparing both proteomic methods [26,45]. Using the shotgun approach, a total of 426 proteins were shared between samples, while 1588 and 1,714 were specifics to SNA and LNA, respectively. Similar protein distributions are found in human [46], buffalo [6], goat [7], and horse [30,31]. Using the same batch of samples for the gel-based 2D-DIGE proteomic approach, 300 out of 2321 total unique spots were detected in both SNA and LNA. Comparative analyses of samples revealed that in SNA, both proteomic approaches showed up-regulations of protein precursors such as plasminogen, nexin-1, inter-alpha-trypsin inhibitor heavy chain H2, and vitronectin playing roles during later follicle stage. For instance, plasminogen [47] and inter-alpha-trypsin inhibitor heavy chain H2 [48,49] appear to act on oocyte competence acquisition through cumulus expansion, while nexin-1 regulates the process [50] and vitronectin contributes to sperm and oocyte linking [51,52]. In LNA, the up-regulation of proteins such as the complement C4 precursor [53] and alpha-1 antichymotrypsin 2 precursor [54] are associated with the inflammatory system in preparation of the ovulation process.

For further biological function analyses, the higher annotation (~88%) of total detected proteins provided meaningful predictions of protein functions through bioinformatics, using gene ontology (GO) terms. Among the shared proteins, only those up-regulated in LNA revealed significantly enriched GO terms. Proteins associated with extracellular localization and blood microparticle were significantly up-regulated in LNA, and datasets contained higher levels of albumin and immunoglobulin as previously reported with FF of other species [6,7,30,31]. In addition, the remarkable increase in enzymes associated with regulatory and inhibitory activities, including serine-type endopeptidases, is expected to control numerous biological, cellular, and metabolic processes occurring throughout folliculogenesis [55]. Numerous proteins were associated with significantly enriched bioprocesses such as blood coagulation (e.g., F12 and PLG), responses to stress and stimulus (e.g., AHSG, F12, HP, and PLG), and inflammation and acute-phase responses (e.g., AHSG, HP). These proteins may participate in cascade of coagulation having a role in follicle growth and oocyte transfer to the oviduct following ovulation [56].

Protein-to-protein interactions (PPI) are of interest in biology because they regulate almost all cellular, metabolic, and biological processes. In the present study, only the LNA protein dataset revealed significant enrichment, and the proteins having the highest number of interactions play important roles on cytoplasmic maturation. Amongst these proteins, plasminogen [47], AMBP [57], and alpha-2-macroglobulin [58] positively influence cumulus expansion, while alpha-2-HS-glycoprotein regulates the binding of spermatozoa on zona pellucida [59], which could influence the incidence of polyspermy in pigs [14]. In addition, the histidine-rich glycoprotein (HRG) protein may act on blood coagulation [60] inhibiting the action of vascular endothelial growth factor [61].

The search for molecular interactions, reactions and relations allowed for the detection of numerous (KEGG) pathways in various datasets (shared and specific proteins in SNA and LNA). Significantly increased pathways associated with SNA (specific and up-regulated) datasets illustrate the importance of cell proliferation and follicle development (Apelin signaling, estrogen signaling, and PI3K-Akt signaling pathways), a critical phase for normal growth follicle [62,63]. In addition, the significant enrichments of “gap junction” and “ECM-receptor interaction” pathways likely indicate high sensitivity of small follicles to stimulus response, cells adhesion, differentiation, proliferation, and apoptosis.

In LNA however, enriched pathways appeared to have a role in the ovulation process. Some constituents of the “focal adhesion” pathway in the specific protein dataset may link to cellular cytoskeleton or membrane receptors to launch signaling events that participate to the ovulation process. Meanwhile, up-regulated proteins in LNA favored the “complement and coagulation cascades” pathway which roles in inflammatory response [64], a required process for ovulation [65] have been reported in FF of several females, including women [66], goats [7], and mares [31].

The last but not the least, we found several male-known proteins in FF from SNA, LNA, or both. These proteins are expected to have potential role in cell-cycle and fertilization. For instance, spermatogenesis-associated protein 22 is required for meiotic progress in germinal cells [67], while izumo sperm–egg fusion protein is essential for gamete recognition and adhesion, forming an intracellular bridge between spermatozoon and oocyte during fertilization [68,69].

## 5. Conclusions

The use of the shotgun method to investigate the follicular fluid proteomes of small and large follicles of porcine ovaries generate larger number of proteins that the gel-based approach. We were able to identify six differentially expressed proteins in both samples, using both proteomics approaches. Numerous biological functions were revealed within each sample, possessing specific differences. Up-regulated proteins in small follicles were directed to the healthy growth of both follicle and oocyte, while those in large follicles were involved in the ovulatory process. Additionally, numerous proteins with roles during fertilization are also reported in the follicular fluid.

## Figures and Tables

**Table 1:** Top 10 enriched gene ontology (GO) terms up-regulated in LNA. No significant GO enrichment was found with up-regulated proteins in the SNA dataset.

Classification	Gene Ontology (GO) Term	Observed	Background	False Discovery
Category	Identification/Description	Gene (*n*)	Gene (*n*)	Rate
Cellular component	GO:0005576/Extracellular region	11	352	9.20 × 10^−9^
	GO:0005615/Extracellular space	5	185	0.00076
	GO:00725615/Blood microparticle	2	5	0.00076
Biological process	GO:0010951/Negative regulation of endopeptidase activity	5	31	6.01 × 10^−6^
Biological process	GO:0043086/Negative regulation of catalytic activity	6	68	6.01 × 10^−6^
	GO:0050790/Regulation of catalytic activity	7	161	6.01 × 10^−6^
	GO:0051336/Regulation of hydrolase activity	6	93	6.01 × 10^−6^
	GO:0065007/biological regulation	11	716	1.75 × 10^−5^
	GO:0048519/negative regulation of biological process	7	280	8.86 × 10^−5^
	GO:0006950/response to stress	6	224	0.00026
	GO:0050789/regulation of biological process	9	648	0.00026
	GO:1901564/organonitrogen compound metabolic process	7	375	0.00037
	GO:0043170/macromolecule metabolic process	7	388	0.00044
Molecular function	GO:0030234/Enzyme regulator activity	7	90	1.72 × 10^−7^
Molecular function	GO:0004857/Enzyme inhibitor activity	6	55	1.98 × 10^−7^
Molecular function	GO:0004866/Endopeptidase inhibitor activity	5	30	3.96 × 10^−7^
	GO:0004867/Serine-type endopeptidase inhibitor activity	3	16	0.0001
	GO:0004252/Serine-type endopeptidase activity	2	21	0.0109
	GO:0005215/Transporter activity	3	90	0.0109

**Table 2:** Reactome pathways in protein datasets. No significant pathways were found for protein specific to LNA.

Reactome Pathway Term		Observed	Background	False Discovery
Identification	Description	Gene (*n*)	Gene (n)	Rate
Up-Regulated Proteins in SNA				
ssc392499	Metabolism of proteins	11	1310	0.0107
Ssc163685	Integration of energy metabolism	3	66	0.0301
ssc597592	Post-translational protein modification	9	1076	0.0301
ssc8957275	Post-translational protein phosphorylation	3	74	0.0301
ssc381426	Regulation of Insulin-like Growth Factor (IGF) transport and uptake by Insulin-like Growth Factor Binding Proteins (IGFBPs)	3	83	0.0307
ssc109582	Hemostasis	5	412	0.0356
ssc1474244	Extracellular matrix organization	4	219	0.0356
ssc163359	Glucagon signaling in metabolic regulation	2	21	0.0356
ssc418597	G alpha (z) signaling events	2	22	0.0356
ssc432040	Vasopressin regulates renal water homeostasis via Aquaporins	2	26	0.0356
Specific Proteins to SNA				
ssc0392499	Metabolism of proteins	78	1310	0.0188
ssc1640170	Cell cycle	35	461	0.0305
ssc5610787	Hedgehog’off state	12	87	0.0305
ssc597592	Post-translational protein modification	65	1076	0.0305
ssc69278	Cell cycle, mitotic	31	398	0.0305
Up-Regulated Proteins in LNA				
SSC-114608	Platelet degranulation	5	78	0.0002
SSC-140837	Intrinsic Pathway of Fibrin Clot Formation	3	13	0.00032
SSC-109582	Hemostasis	7	412	0.0014
SSC-75205	Dissolution of Fibrin Clot	2	6	0.0032
SSC-140875	Common Pathway of Fibrin Clot Formation	2	14	0.0119
SSC-977606	Regulation of Complement cascade	2	32	0.0481

**Table 3:** Top enriched Kyoto Encyclopedia of Genes and Genomes (KEGG) pathways in up-regulated and specific proteins in SNA and LNA datasets.

Sample Type	KEGG Pathway Identification	Observed	Background	False
(Dysregulation)	Identification/Description	Gene Count	Gene Count	Discovery Rate
SNA-specific	ssc04540/Gap junction	11	73	0.0418
	ssc04915/Estrogen signaling pathway	13	109	0.0418
	Ssc04971/Gastric acid secretion	10	60	0.0418
LNA-specific	ssc04974/Protein digestion and absorption	13	70	0.0048
LNA-specific	ssc04510/Focal adhesion	18	163	0.0341
	ssc05165/Human papillomavirus infection	24	259	0.041
Up-regulated SNA (↑SNA ↓LNA)	ssc04512/ECM-receptor interaction	3	66	0.0184
Up-regulated SNA (↑SNA ↓LNA)	ssc04713/Circadian entrainment	3	72	0.0184
	ssc05165/Human papillomavirus infection	5	259	0.0184
	ssc04371/Apelin signaling pathway	3	108	0.0358
	ssc05200/Pathways in cancer	5	428	0.0358
	ssc04151/PI3K-Akt signaling pathway	4	284	0.0439
Up-regulated LNA (↑LNA ↓SNA)	ssc04610/Complement and coagulation cascades	7	73	3.33 × 10^−8^
Up-regulated LNA (↑LNA ↓SNA)	ssc05150/Staphylococcus aureus infection	4	39	8.17 × 10^−5^

Dysregulated proteins correspond to upregulation (↑) and downregulation (↓) in SNA or LNA samples.

**Table 4:** Examples of dysregulated proteins detected with both shotgun and gel-based proteomics.

Protein		SNA		LNA	
Name	GI	Shotgun	Gel-Based	Shotgun	Gel-Based
Complement C4 precursor	178056221	↓	↓	↑	↑
Alpha-1-antichymotrypsin 2 precursor	47523270	↓	↓	↑	↑
Plasminogen precursor	113205806	↑	↑	↓	↓
Nexin-1 precursor	47523638	↑	↑	↓	↓
Vitronectin precursor	55741847	↑	↑	↓	↓
Inter/alpha-trypsin inhibitor heavy chain H2 precursor	47522678	↑	↑	↓	↓

Arrows indicate significant (*p* < 0.05) upregulation (↑) and downregulation (↓) between SNA and LNA samples.

## Supplementary Materials

The following are available online at https://www.mdpi.com/2079-7737/9/5/101/s1, Table S1: Proteome datasets.

## Abbreviations

AHSG	Alpha-2-HS-glycoprotein
A2M	Alpha-2-macroglobulin
BP	Biological process
CC	Cellular component
HRG	Histidine-rich glycoprotein
E2	Estradiol
FF	Follicular fluid
GO	Gene ontology
HRG	Histidine-rich glycoprotein
KEGG	Kyoto Encyclopedia of Genes and Genomes
LFF	Large follicular fluid
LNA	Large non-atretic
MF	Molecular function
NA	Non-atretic
PLG	Plasminogen
PPI	Protein-to-protein interactions
SFF	Small follicular fluid
SNA	Small non-atretic
2D-DIGE	Two Dimension-Differential In Gel Electrophoresis
